# Comparing life expectancy and health-adjusted life expectancy by body mass index category in adult Canadians: a descriptive study

**DOI:** 10.1186/1478-7954-11-21

**Published:** 2013-11-19

**Authors:** Colin Steensma, Lidia Loukine, Heather Orpana, Ernest Lo, Bernard Choi, Chris Waters, Sylvie Martel

**Affiliations:** 1 Public Health Agency of Canada, 200, boulevard René-Lévesque Ouest, Montréal, QC H2Z 1X4, Canada; 2 Public Health Agency of Canada, 785 Carling, Ottawa, ON K1A 0K9, Canada; 3 Institut national de santé publique du Québec, 190, boulevard Crémazie Est, Montréal, QC H2P 1E2, Canada

**Keywords:** Overweight, Obesity, Underweight, Body mass index, Life expectancy, Health expectancy, Mortality, Health-related quality of life

## Abstract

**Background:**

While many studies have examined differences between body mass index (BMI) categories in terms of mortality risk and health-related quality of life (HRQL), little is known about the effect of body weight on health expectancy. We examined life expectancy (LE), health-adjusted life expectancy (HALE), and proportion of LE spent in nonoptimal (or poor) health by BMI category for the Canadian adult population (age ≥ 20).

**Methods:**

Respondents to the National Population Health Survey (NPHS) were followed for mortality outcomes from 1994 to 2009. Our study population at baseline (n=12,478) was 20 to 100 years old with an average age of 47. LE was produced by building abridged life tables by sex and BMI category using data from the NPHS and the Canadian Chronic Disease Surveillance System. HALE was estimated using the Health Utilities Index from the Canadian Community Health Survey as a measure of HRQL. The contribution of HRQL to loss of healthy life years for each BMI category was also assessed using two methods: by calculating differences between LE and HALE proportional to LE and by using a decomposition technique to separate out mortality and HRQL contributions to loss of HALE.

**Results:**

At age 20, for both sexes, LE is significantly lower in the underweight and obesity class 2+ categories, but significantly higher in the overweight category when compared to normal weight (obesity class 1 was nonsignificant). HALE at age 20 follows these same associations and is significantly lower for class 1 obesity in women. Proportion of life spent in nonoptimal health and decomposition of HALE demonstrate progressively higher losses of healthy life associated with lowered HRQL for BMI categories in excess of normal weight.

**Conclusions:**

Although being in the overweight category for adults may be associated with a gain in life expectancy as compared to normal weight adults, overweight individuals also experience a higher proportion of these years of life in poorer health. Due to the descriptive nature of this study, further research is needed to explore the causal mechanisms which explain these results, including the important differences we observed between sexes and within obesity subcategories.

## Background

As rates of overweight and obesity continue to climb in Canada and many other developed countries, curbing and reducing these rates has become a long-term goal for public health practitioners in these countries [1]. Underweight adults are also at risk for negative health consequences including elevated mortality [2]. One approach that can aid in the evaluation of a population’s health according to body weight category is the estimation of health expectancy by body mass index (BMI). Health expectancy measures such as health-adjusted life expectancy (HALE) combine life expectancy (LE) with a measure of health-related quality of life (HRQL) or disability to create an indicator for assessing the combined effects of health and mortality, which is expressed in an intuitive measure similar to that of life expectancy [3]. Furthermore, in populations where life expectancy is increasing, health expectancies can be used to monitor whether the proportion of life spent in health is increasing (compression of morbidity) or decreasing (expansion of morbidity) due to a particular health problem such as insufficient or excess body weight [4].

Certain associations have been demonstrated between the body weight categories of underweight, overweight, and obese (including its two subcategories: moderately obese and severely obese) and premature mortality. Recent national population-based studies in Canada and the United States [2,5,6] have demonstrated that being in the underweight or severely obese BMI category is associated with an increased risk of mortality amongst adults in the general population when compared to their peers in the normal weight BMI category (18.5 ≤ BMI < 25 kg/m^2^). These same studies also demonstrated a decreased risk of mortality for those in the overweight category when compared to those in the normal weight category: a result which was supported by a recent meta-analysis of all-cause mortality for overweight and obesity relative to normal weight, which included 97 studies with a combined sample size of more than 2.88 million individuals and more than 270,000 deaths [7]. Other studies have demonstrated an increased risk of mortality with overweight and lower levels of obesity. However, these studies tended to be based either on more restricted populations that exclude older adults, as well as certain socio-economic status and ethnic groups which may have a greater number of competing mortality risks as compared to the general population [8-10]. Additionally, some of these study populations consist of less-recent cohorts [11,12] where follow-up occurred during time periods when mortality risk for excess weight may have been greater [6].

When the effects of BMI on health-related quality of life are observed, the literature for population-level studies [13-15] generally demonstrate that for women, there is a consistent decrease in HRQL across excess weight categories. For men, overweight and moderate obesity were associated with higher HRQL in one study [14], while another study [15] reported an optimal HRQL for men with a BMI of 26 as opposed to a BMI of 24.5 for women. A study of HRQL by BMI category in US adults found that three of six commonly-used HRQL indices produced significantly lower HRQL results for obese men as compared to normal weight men [13]. The two studies [14,15] which addressed underweight demonstrated that this weight category is associated with lower HRQL in both sexes, although this association appears to be age-dependent for women [14].

While these studies provide insight into health losses due to premature mortality and reduced HRQL, there are a limited number of studies that examine how BMI impacts on both of these two measures in the form of health expectancy. Four studies have examined health expectancy for multiple BMI categories [16-19]. When stratified by sex, the estimates for health expectancy in these studies range from small gains for overweight women and negligible losses for men in the excess weight categories, to considerable loss of health expectancy for overweight women and obese men and women. Several of these studies have one or more limitations which make it difficult to assess health expectancy results by BMI category at the population level. Two of these studies [17,18] restricted their study populations to a subgroup of those 55 years and older, as well as to white Americans and a single city in the Netherlands, respectively. Another of these studies [19] based its relative risks for obesity-related morbidity and mortality on studies using populations other than the study population, which may lead to biased estimates [20]. In addition, none of these studies presented complete health expectancy results by obesity subgroup (i.e., moderate and severe obesity), despite the important variations in mortality risk and loss of HRQL observed in these subcategories in previous studies. Health expectancy for those in the underweight category has also not been explored to date.

The objective of this study was to address the above-mentioned gaps by exploring differences in LE and HALE for each of the World Health Organization BMI categories, including two obesity subclasses (class 1 and class 2+), using a representative sample of the Canadian adult population aged 20 years and older. We also assessed the specific contribution of HRQL to losses in HALE by using two measures: the difference between LE and HALE proportional to LE and the decomposition of HALE into each of its mortality and HRQL components.

## Methods

### Data sources

We used National Population Health Survey (NPHS) data for estimation of mortality attributable to body weight status, Canadian Community Health Survey (CCHS) data for BMI prevalence and HRQL estimation, and Canadian Chronic Disease Surveillance System (CCDSS) data to estimate total mortality rates. The study was limited to the adult (20 years old and over) Canadian population.

Both NPHS and CCHS are conducted by Statistics Canada and are designed to collect information on the health and the determinants of health of the Canadian population. In both surveys, the samples are weighted to be representative of the Canadian population. NPHS is a longitudinal survey while the CCHS is cross-sectional. The methodology of these surveys has been described in detail elsewhere [21,22] and are briefly summarized here.

The first cycle of the NPHS data collection took place in 1994/1995 and follow-up with this cohort continued every second year thereafter until the ninth and final cycle in 2010/2011. We used eight cycles of the data covering the period from 1994 to 2009. The target population of the NPHS household component includes household residents of all ages in the 10 Canadian provinces in 1994/1995 excluding persons living on Indian reserves and Crown lands, residents of health institutions, full-time members of the Canadian Forces living on Canadian Forces bases, and residents of some remote areas in Ontario and Quebec. The initial sample size was 20,095, and the initial response rate was 86% which lead to 17,276 respondents, of whom 15,805 agreed to share their information with Health Canada and the Public Health Agency of Canada. Mortality follow-up occurred regardless of response status, and thus there is little loss to follow-up with mortality as the studied end point. The survey includes self-reported height and weight as well as information about health status and health determinants. Eight cycles, or 14 years of follow-up, were used in this study to estimate the mortality Hazard Ratio (HR) according to BMI category at baseline. The study population included 12,478 participants 20 years old and over. Participants with missing BMI information at baseline (n = 571) were also excluded, leading to a sample size of 11,907 for the present study with an age range of 20 to 100 years old and an average age of 47 in 1994/1995.

The CCHS is a nationally representative sample of Canadians 12 years of age and over dwelling in households, with the same exclusions of participants as those found in the NPHS. Data are available from 2000 and, since 2007, are collected on an ongoing basis. Prior to 2007, data collection occurred every two years over a 12-month period. In order to reduce the variability in our estimates, we combined the first three CCHS cycles, which span the years 2000–2005. The sample size generated by combining these three cycles for BMI prevalence estimation was 317,996, with an age range of 20 to 104. The household-level response rate was between 84.9% and 91.4%, and the individual-level response rate was between 91.9% and 92.9% during this period. Two different methods are recommended for combining data from different survey cycles: the separate approach and the pooled approach [23]. The choice of approach is based, amongst other things, on the degree to which the parameter being measured remains constant between cycles. The age-sex-specific BMI prevalences were estimated based on the separate approach, in which a simple average of estimates from each CCHS cycle being combined is calculated. Since the age-sex-specific Health Utilities Index (HRQL measure explained in more detail below) estimates were more stable across cycles, the pooled approach was used in which the microdata from each cycle are combined into a single sample.

The CCDSS is a national collaborative network of provincial and territorial chronic disease surveillance systems that collect administrative health care data [24]. The CCDSS data include death and population counts by sex and five-year age groups and health information such as the prevalence and incidence of selected chronic diseases. The CCDSS represents almost the entire Canadian population, excluding full-time members of the Canadian Forces, the Royal Canadian Mounted Police, and individuals in federal correctional facilities. Total mortality rates were estimated from CCDSS data for the period of 2000–2005 by sex and five-year age groups. Ethical approval was not required as this study was based on analysis of secondary data and no experimental research on humans was carried out.

### Indicators

Self-reported weight and height were used to calculate BMI in the NPHS and CCHS by sex and age group. BMI categories were defined in the study as underweight (BMI < 18.5 kg/m^2^), normal weight (18.5 ≤ BMI < 25 kg/m^2^), overweight (25 ≤ BMI < 30 kg/m^2^), obese class 1 (30 ≤ BMI < 35 kg/m^2^), and obese class 2+ (BMI ≥ 35 kg/m^2^). A sensitivity analysis was performed to evaluate the impact of using self-reported BMI as compared to BMI corrected for self-report bias [25].

The measure of HRQL used in the present study was the Health Utilities Index (HUI) Mark 3 [26], which has been used in previous studies to calculate HALE [27]. HUI is a preference-based measure that defines health states according to eight attributes (vision, hearing, speech, ambulation, dexterity, emotion, cognition, and pain), with five or six levels ranging from normal to severely limited functioning for each. Single attribute utility scores range from 0.0 (lowest level of functioning) to 1.0 (full functional capacity). The eight attributes are combined into a single score using the multi-attribute utility function:

(1) $$u=1.371*\left(b_{1}*b_{2}*b_{3}*b_{4}*b_{5}*b_{6}*b_{7}*b_{8}*\right)-0.371,$$

where *u* is HUI and *b*_*i*_ represents the i-th attribute utility score [28].

The overall scores on the HUI range from -0.36 (the worst possible HUI health state) through 0.0 (death) to 1.0 (perfect health). From a societal perspective, some health states are considered worse than death, and consequently are assigned negative scores. Details are described elsewhere [28] on how the utility scores are derived from preference scores for individual attributes. Differences of 0.03 or more in overall HUI scores and 0.05 or more in single-attribute utility scores are considered to be clinically important [26]. CCHS data from combined cycles were used to estimate HUI by age group, sex, and BMI category.

### Calculating mortality

The relative risk of dying was approximated using the estimated hazard ratios (HR) for underweight, overweight, obese class 1, and obese class 2+ BMI categories as compared to the normal weight category. Data from the NPHS provided BMI and age values at baseline as well as mortality during follow-up. A discrete-time proportional hazard model using a complementary log-log function (clog-log) was used to estimate HR. The model was adjusted by BMI category, sex, and age group. The data were categorized into two age groups (20–64 and ≥65) with the assumption that the risk was constant within each group. The linearity assumption was supported and goodness of fit was confirmed using the Hosmer-Lemeshow test.

By taking into account BMI prevalence by sex and five-year age group and the relative risk of dying for each BMI category relative to the normal weight category by sex and for two age groups, the age-specific mortality rates for the total Canadian population were partitioned into mortality rates by BMI class, sex, and five-year age group. The formulae used to determine mortality rates are available in an Additional file 1 to this article.

### Calculation of LE and HALE

The Chiang method [29] was used to generate period (2000–2005) life tables by BMI categories and sex using 14 standard age groups (20–24.9, 25–29.9 …, 80–84.9, ≥85 years). Closure of life tables was done by fitting a Gompertz function to the last open-ended ≥85 age interval, as described by Hsieh [30]. The Sullivan method [31] was applied for the HALE calculation. This method has been demonstrated to be an unbiased and consistent estimator of health expectancy [32]. According to this method the “life-years lived” were adjusted by the HUI.

(2) $$L_{x}^{\prime }=L_{x}*\mathrm{HU}I_{x}$$

Where $L_{x}^{\prime }$ are adjusted life-years lived in age-interval x, L_x_ are life-years lived in age-interval x, and HUI_x_ is Health Utilities Index Mark 3 for people in age-interval x.

The variance and 95% confidence intervals for LE and HALE were estimated using the bootstrap methodology [33]. The bootstrapping approach we used involves applying the bootstrap weights calculated by Statistics Canada through a resampling of the NPHS and CCHS surveys. These account for the complex survey designs by producing new sets of survey weights with each iteration. 250,000 HR, BMI prevalence, and HUI combinations were generated, with each combination leading to a set of HALE by BMI results. Z-tests were used to test the statistical significance of the differences in LE and HALE between BMI categories.

In order to assess the proportion of life expectancy spent in a less-than-optimal (or poor) state of health (P), the difference between LE and HALE was divided by LE:

(3) $$P=\frac{\mathrm{LE}-\mathrm{HALE}}{\mathrm{LE}}$$

### Subanalysis of mortality and HRQL components

The Nusselder decomposition (i.e., partitioning) method [34] was applied to quantify the difference in HALE attributable to differences in premature mortality and differences in loss of HRQL between BMI categories. This technique is based on the Sullivan method and is an extension of the decomposition method for life expectancy developed by Arriaga [35]. For each age group, the change in HALE between the comparison groups (Δ*HALE* = *L*_x1_*HUI*_1_ – *L*_x2_*HUI*_2_, where *L*_*x*2_ refers to the number of years lived by the persons in the normal weight reference category for age interval *x* and *L*_*x*1_ refers to the number of years lived by the persons in the comparison BMI category for age interval *x*) is partitioned into the following components:

(4) $$\begin{array}{l} \Delta \mathrm{HALE}=\Delta \mathrm{MORB}+\Delta \mathrm{MORT}\\ \;=\frac{L_{X1+}L_{X2}}{2}\Delta \mathrm{HUI}+\frac{\mathrm{HU}I_{1}+\mathrm{HU}I_{2}}{2}\Delta L_{X} \end{array}$$

The first component (Δ*MORB*) estimates changes due to HRQL and the second one (Δ*MORT*) estimates changes due to mortality. The age specific components are then summed to give the HRQL and mortality decomposition.

All calculations and statistical analyses were conducted using SAS version 9.2.

## Results

### BMI prevalence and HRQL by BMI category

Age-sex-specific BMI category prevalences and HUI scores by BMI category are shown in Table 1. Normal weight is the most prevalent category for women in all age categories studied, whereas for men, this was only the case for those 20–34 years old and 75 years and older: overweight was the most prevalent category for men in the other age groups. The prevalences of underweight and obesity class 2+ are relatively low in comparison to the other BMI groups. For HUI in women, normal weight is generally associated with the highest HRQL, followed closely by overweight, underweight, obesity class 1, and obesity class 2+. HUI scores for women ≥20 years old are significantly lower for all BMI categories when compared to women in the normal weight category (p < 0.05). For men, overweight and normal weight are associated with the highest HRQL. Obesity class 1 is associated with a lower level of HRQL, although values converge with those of overweight and normal weight men in middle age (40–55 years old). Obesity class 2+ men have the second lowest level of HRQL, with underweight men having the lowest. Men ≥20 years old in the underweight category and in both obesity subclasses have significantly lower HUI scores (p < 0.05).

**Table 1:** BMI prevalence by category and HRQL by BMI category, sex, and age group, Canada, 2000-2005

	**BMI category prevalence (%)**					**HRQL by BMI category (HUI score)**				
**Females**										
**Age group (years)**	**Under-weight**	**Normal weight**	**Over-weight**	**Obesity class 1**	**Obesity class 2+**	**Under-weight**	**Normal weight**	**Over-weight**	**Obesity class 1**	**Obesity class 2+**
20-24	9.9	67.9	14.8	5.1	2.3	0.88	0.92	0.90	0.88	0.81
25-29	6.9	61.9	19.4	7.6	4.2	0.92	0.92	0.90	0.89	0.90
30-34	4.4	59.3	23.0	8.9	4.4	0.89	0.91	0.89	0.88	0.81
35-39	4.2	59.1	23.1	9.0	4.6	0.92	0.91	0.91	0.87	0.83
40-44	3.4	57.3	25.2	9.5	4.6	0.86	0.90	0.88	0.86	0.80
45-49	2.5	53.7	28.2	10.3	5.4	0.85	0.89	0.86	0.83	0.80
50-54	1.9	46.9	32.4	13.1	5.8	0.79	0.88	0.86	0.84	0.74
55-59	1.7	43.5	34.9	13.7	6.2	0.82	0.88	0.87	0.79	0.76
60-64	2.0	42.2	35.8	14.4	5.6	0.80	0.88	0.84	0.81	0.74
65-69	2.4	43.0	36.6	13.1	4.9	0.84	0.87	0.86	0.81	0.69
70-74	3.2	43.4	34.9	13.7	4.8	0.76	0.84	0.83	0.77	0.62
75-79	4.3	46.7	34.4	11.5	3.2	0.75	0.79	0.78	0.69	0.55
80-84	5.9	51.1	31.1	9.7	2.2	0.62	0.74	0.69	0.61	0.63
≥85	9.2	56.9	25.5	6.8	1.6	0.55	0.60	0.64	0.54	0.36
All ages (≥20)	4.1	53.6	27.4	10.3	4.6	0.85*	0.89	0.86*	0.82*	0.77*
**Males**										
**Age group (years)**	**Under-weight**	**Normal weight**	**Over-weight**	**Obesity class 1**	**Obesity class 2+**	**Under-weight**	**Normal weight**	**Over-weight**	**Obesity class 1**	**Obesity class 2+**
20-24	2.6	60.4	27.9	6.5	2.6	0.84	0.91	0.92	0.89	0.86
25-29	1.6	49.1	35.0	10.8	3.5	0.82	0.92	0.92	0.91	0.88
30-34	0.9	42.3	40.1	13.1	3.6	0.86	0.92	0.93	0.91	0.87
35-39	0.8	39.3	43.3	12.9	3.7	0.81	0.90	0.91	0.92	0.84
40-44	0.8	38.1	44.5	13.1	3.5	0.83	0.90	0.91	0.91	0.86
45-49	0.6	36.5	44.1	14.7	4.2	0.70	0.89	0.90	0.91	0.84
50-54	0.5	34.2	44.9	15.5	4.8	0.73	0.87	0.89	0.88	0.84
55-59	0.5	33.0	45.4	16.5	4.6	0.72	0.89	0.87	0.86	0.81
60-64	0.8	34.1	45.5	15.6	4.0	0.81	0.86	0.87	0.85	0.76
65-69	0.9	35.8	44.8	14.7	3.8	0.74	0.88	0.86	0.83	0.76
70-74	1.1	38.8	45.6	11.8	2.7	0.74	0.86	0.85	0.79	0.78
75-79	1.5	43.4	43.3	10.1	1.7	0.73	0.80	0.79	0.81	0.75
80-84	2.8	49.7	38.1	7.9	1.5	0.54	0.74	0.73	0.72	0.55
≥85	2.5	58.6	32.8	5.5	0.5	0.52	0.64	0.61	0.48	0.57
All ages (≥20)	1.1	41.1	41.4	12.8	3.6	0.79*	0.89	0.89	0.88*	0.83*

Source of data: Canadian Community Health Survey, combined cycles 2000–2001, 2003, and 2005, Statistics Canada.

^*^p < 0.05: statistically significant difference vs. normal weight (significance tests for HUI score, all ages, by sex only).

During the 14-year follow-up period, 2113 deaths were observed in our study population. We observe a significantly increased risk of mortality among the underweight (age-sex-adjusted HR 1.49, 95% CI: 1.17, 1.90) and obesity class 2+ (age-sex-adjusted HR 1.42, 95% CI: 1.16, 1.72) categories, as compared to the normal weight category. We observe a significantly reduced risk of mortality among the overweight (age-sex-adjusted HR 0.69, 95% CI: 0.62, 0.78) and obesity class 1 (age-sex-adjusted HR 0.84, 95% CI: 0.72, 0.98) categories, as compared to the normal weight category. When HRs are stratified by age group (20–64 and ≥65 years old, results not shown), significantly elevated risks for mortality are observed for underweight individuals 65 years of age or older, as well as for those aged 20 to 64 years old in the obesity class 2+ category (p < 0.05). Risk for mortality is significantly decreased for those 65 years or older in the overweight and obesity class 1 categories (p < 0.05). The direction of the mortality risk association is the same between age groups for each BMI category with the exception of underweight. The sensitivity analysis performed to evaluate the impact of using self-reported BMI compared to BMI corrected for self-report bias (results not shown) demonstrates that HRs recalculated using the corrected BMI only marginally change the estimates of mortality risk by BMI category. However, the obesity class 1 HR is no longer statistically significant at the p < 0.05 threshold when the correction factor is implemented.

Life expectancy at 20 years of age is lowest for Canadian men and women in the underweight and obesity class 2+ categories, whereas the highest LE at this age is found for those in the overweight, obesity class 1, and normal weight categories (Table 2).

**Table 2:** Life Expectancy (LE), Health-Adjusted Life Expectancy (HALE), and differences in LE and HALE at age 20 by BMI category and sex, Canada, 2000-2005

**Females**					
	**Underweight**	**Normal weight**	**Overweight**	**Obesity class 1**	**Obesity class 2+**
LE	58.7^*^	62.8	66.5^*^	64.6	59.3^*^
(95% CI)	(56.3, 61.6)	(62.6, 63.0)	(65.5, 67.5)	(63.0, 66.2)	(56.9, 61.3)
HALE	48.5^*^	54.1	55.6^*^	51.4^*^	44.1^*^
(95% CI)	(46.7, 50.6)	(53.8, 54.4)	(54.8, 56.5)	(50.2, 52.8)	(42.6, 45.8)
LE – HALE	10.2	8.7	10.9	13.2	15.2
[LE - HALE]/LE	0.174	0.139	0.164	0.204	0.256
**Males**					
	**Underweight**	**Normal weight**	**Overweight**	**Obesity class 1**	**Obesity class 2+**
LE	53.0^*^	57.2	61.0^*^	59.1	53.5^*^
(95% CI)	(50.3, 56.1)	(56.8, 57.6)	(60.2, 61.9)	(57.5, 60.6)	(51.1, 55.6)
HALE	41.0^*^	50.0	52.9^*^	50.4	43.8^*^
(95% CI)	(38.3, 43.6)	(49.6, 50.4)	(52.2, 53.7)	(49.2, 51.6)	(42.0, 45.6)
LE – HALE	12.0	7.2	8.1	8.7	9.7
[LE - HALE]/LE	0.226	0.126	0.133	0.147	0.181

95% confidence intervals based on bootstrapping results.

^*^p < 0.01: statistically significant difference vs. normal weight (significance tests on first two lines for each sex only).

Sources of data: National Population Health Survey, Cycles 1–8 (1994/1995-2009), Statistics Canada; Canadian Community Health Survey, combined cycles 2000–2001, 2003, and 2005, Statistics Canada; Canadian Chronic Disease Surveillance System, 2000–2005, Public Health Agency of Canada.

Health-adjusted life expectancy at 20 years of age for Canadian women is lowest in the obesity class 2+ category, followed by those in the underweight and obesity class 1 categories. Women at this age in the overweight category have the highest HALE, followed by normal weight. For 20-year-old men, those in the underweight and obesity class 2+ categories have the lowest HALE estimates, while those classified as overweight exhibit the highest estimates, followed by obesity class 1 and normal weight.

Results for the difference between LE and HALE for Canadians at age 20 (last two rows of Table 2) show the average cumulative amount of time spent in less than optimal health both in terms of absolute number of years and as a proportion of remaining life expectancy. Differences in LE and HALE for women range from 8.7 years for normal weight up to a high of 15.2 years for women in the obesity class 2+ category. Meanwhile, underweight women spend a greater amount of time in nonoptimal health than normal weight women but spend a lesser amount of time in this state than women in the excess weight categories. Similar trends for women in each BMI category are observed when we examine life spent in less than optimal health as a proportion of life expectancy (represented as (LE-HALE)/LE), the sole exception being underweight women, who have a greater proportion of LE spent in nonoptimal health than do overweight women. When compared to women, men have smaller differences in life years spent in nonoptimal health between the normal and excess weight categories: from 7.2 years in normal weight to 9.7 years in obesity class 2+. Among men, those in the underweight category have the highest absolute number of years spent in poor health with 12.0 years. These trends persist for men when calculated as a proportion of LE.

**Figure 1:**
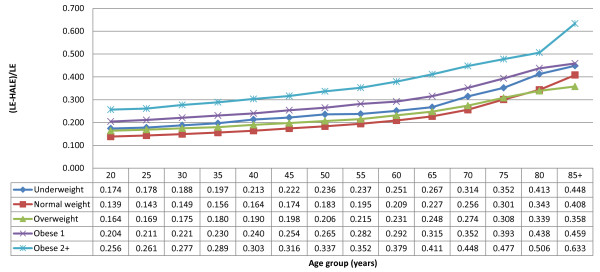
**Proportion of life expectancy spent in nonoptimal health by age group, females, Canada, 2000–2005.** Sources of data: National Population Health Survey, Cycles 1–8 (1994/1995-2009), Statistics Canada; Canadian Community Health Survey, combined cycles 2000–2001, 2003, and 2005, Statistics Canada; Canadian Chronic Disease Surveillance System, 2000–2005, Public Health Agency of Canada.

**Figure 2:**
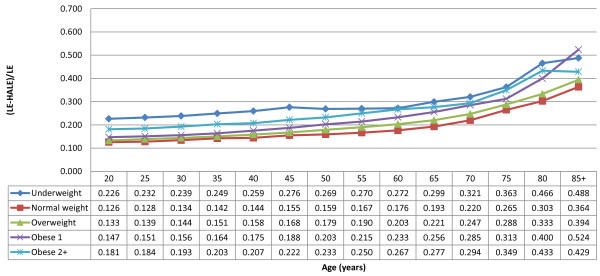
**Proportion of life expectancy in nonoptimal health by age group, males, Canada, 2000–2005.** Sources of data: National Population Health Survey, Cycles 1–8 (1994/1995-2009), Statistics Canada; Canadian Community Health Survey, combined cycles 2000–2001, 2003, and 2005, Statistics Canada; Canadian Chronic Disease Surveillance System, 2000–2005, Public Health Agency of Canada.

When the proportion of LE spent in nonoptimal health is examined by age group (Figures 1 and 2), we see that the differences between BMI categories observed in Canadian women at age 20 remain largely the same across age groups. Obese and underweight women at all ages demonstrate higher proportions of life spent in nonoptimal health compared to normal weight women, while this proportion is also higher amongst overweight women up to the age of 75. However, for men there are some notable differences in the older age groups. Although the general pattern persists (higher proportion of LE spent in nonoptimal health amongst men of excess weight and especially underweight men when compared to normal weight), there is an increasingly greater proportion of LE spent in nonoptimal health for men in the obesity class 1 category as age increases: from a proportion that is 1.17 times higher than normal weight men at age 20 to 1.32 times higher at age 60 and 1.44 times higher at age 85 and older.

When the differences in HALE at age 20 between a given BMI category and the reference normal weight category are broken down (i.e., decomposed) into their HRQL and mortality components (Table 3) for females, there is a progressive loss of HALE due to loss of HRQL as BMI increases beyond normal weight, ranging from a loss of one year in health expectancy in overweight to a loss of almost eight years for women in obese class 2+. In contrast, the contribution of mortality to HALE losses is not seen until obese class 2. When decomposing HALE differences for males at age 20, we see much smaller losses in health expectancy due to HRQL losses (from almost no loss in HALE in overweight to a loss of 3.5 years in obese class 2+), though the same general mortality trends are similar to those of females. Gains in HALE for overweight men and women are entirely accounted for by the contribution of reduced mortality. For underweight, both sexes show significant, large losses in HALE and these losses are seen in both mortality and HRQL components. Underweight males, however, appear to experience a greater burden, which is seen predominantly in the HRQL component.

**Table 3:** Contribution of Mortality and HRQL components to differences in HALE at age 20 between BMI categories (given category vs. normal weight category), by sex, Canada, 2000-2005

	**Females**					**Males**				
	**Under-weight**	**Normal weight**	**Over-weight**	**Obesity class 1**	**Obesity class 2+**	**Under-weight**	**Normal weight**	**Over-weight**	**Obesity class 1**	**Obesity class 2+**
HALE difference: [HALE(i) -HALE(n)]^a^	-5.6*	REF	1.5*	-2.7*	-10.0*	-9.0*	REF	2.9*	0.4	-6.2*
Difference due to premature mortality	-2.8	REF	2.6	1.2	-2.2	-3.0	REF	2.9	1.3	-2.7
Difference due to loss of HRQL	-2.7	REF	-1.0	-3.8	-7.8	-5.9	REF	0.0	-1.0	-3.5

Based on decomposition technique developed by Nusselder and Looman (2004) and Arriaga et al. (1984).

HALE (i) = HALE for indicated BMI category; HALE(n) = HALE for normal BMI category (reference).

*p < 0.01 HALE difference: statistically significant difference vs. normal weight (significance tests on first line only).

^a^Some discrepancies in HALE difference and component difference are due to rounding.

Sources of data: National Population Health Survey, Cycles 1–8 (1994/1995-2009), Statistics Canada; Canadian Community Health Survey, combined cycles 2000–2001, 2003, and 2005, Statistics Canada; Canadian Chronic Disease Surveillance System, 2000–2005, Public Health Agency of Canada.

## Discussion

This study estimated life expectancy and health adjusted life expectancy by BMI category for Canadian men and women, age 20 and over, during the period 2000–2005. Our findings indicate that there are important health expectancy differences between BMI categories, as well as between the sexes for those in the lower excess weight categories. When estimated at 20 years of age, both sexes have significant losses of LE and HALE in the underweight and higher obesity classes and significant gains in LE and HALE in the overweight category when compared to those in the normal weight category. Women and men at 20 also demonstrate progressively higher proportions of life spent in nonoptimal health in excess weight categories as compared to those of normal weight.

Women and men in the obese class 1 category do not share the same HALE experience at 20: the former have a significantly lower HALE while there is no effect for the latter. Furthermore, men in the obese class 1 category saw progressive increases in the proportion of life spent in nonoptimal health with each successive age group that were more substantial than those of normal weight men, while women in this BMI category experienced increases proportional to normal weight women in all age groups. Finally, although overweight men and women both had significantly higher HALE at age 20, overweight women experienced a larger increase in proportion of life spent in nonoptimal health compared to normal weight women, whereas this increase was relatively small when comparing normal weight and overweight men.

This study adds to the current literature on life expectancy and health expectancy by BMI category by using available datasets to estimate age-sex-specific mortality and HRQL by BMI category in Canada. When our results are compared to those of the only other study that examined health expectancy by BMI category using a representative sample of populations at the country level [16], we found similar patterns in life expectancy and proportion of life spent in nonoptimal health (measured as LwD/LE in this other study, where LwD is Life with Disability) amongst normal weight, overweight, and obese men and women. Ours is the first study to our knowledge to report on health expectancy for the obesity subcategories and for the underweight category. When the WHO BMI category for obesity was broken down into subclasses, we observed important variations in HALE by sex. Majer et al. [16] estimated the hazard rates of various disability states by obesity subcategory in a sensitivity analysis and also found heterogeneity in this weight category: those in the obese class 2+ category were significantly less likely to recover from disability compared to participants of normal weight, while those in the obese class 1 category demonstrated no significant difference for recovery. Other population-based studies examining mortality risk and years of life lost by obesity subcategory [2,5,6,36] demonstrate a similar trend of modest to negative risk for premature mortality in the lower obesity class coupled with more significant risks in the higher classes. This trend has also been observed for men with respect to loss of HRQL, although women tend to have a more important loss of HRQL as BMI increases [14]. With respect to underweight individuals, the observed results are consistent with what was expected based on the evidence regarding both mortality and HRQL among this group. Underweight may be associated with malnutrition, sarcopenia, low-grade inflammation, and frailty, which are each associated with mortality risk and decreased quality of life [37].

The results we observed for LE by BMI category appear to support those found in the recent meta-analysis of all-cause mortality for overweight and obesity relative to normal weight in the general population [7]. This meta-analysis and individual studies with similar results have generated much discussion about the so-called “obesity paradox” where lower mortality risk is associated with those in the overweight BMI category and where there is no difference in mortality risk associated with the obesity class 1 category compared to those in the normal weight category. Some of the potential reasons proposed for these seemingly counterintuitive results are addressed in the limitations section below: imperfect nature of BMI as a predictor of metabolic risk; BMI being measured solely at baseline and thus not accounting for the effect of body weight changes over time; confounding due to pre-existing illness at baseline; use of self-reported height and weight; and issues around proper control for tobacco use and other potentially modifying factors in the analysis. Other issues of importance not addressed here include: heterogeneity of mortality risk in the BMI normal weight category (i.e., those with a BMI between 18.5 and 22 have been shown to have higher mortality risk); better management of risk factors in overweight and obese clients by the health care community; and the possible benefit of having some adipose reserve during periods of acute catabolic illness [38].

In the absence of a sufficiently large dataset with a measure of BMI and mortality follow-up, our approach of estimating Canadian HR for BMI mortality and combining this with death data to create age-sex-specific mortality rates by BMI category represents a feasible alternative method. However, the hazard ratios used to produce age-sex-specific BMI mortality are based on height and weight assessments made among the Canadian adult household population in 1994/1995. It is possible that the relationship between BMI and mortality has changed in subsequent cohorts, which would have an impact on estimates of LE and HALE. Future research should be conducted to confirm our results by using national mortality follow-up of early Canadian Community Health Survey cycles in order to determine more recent and stable mortality rates by BMI category.

While our study was based on a representative sample of the Canadian population, certain subpopulations were excluded from the NPHS and CCHS, most notably those in long-term care institutions. It is possible that risks for mortality and loss of HRQL are greater among those living in long-term care institutions and, as such, these individuals may not have the same LE and HALE profile as that of the community-dwelling population used in our study.

We did not assess BMI at multiple intervals during follow-up, which could lead to misclassification of mortality risk for subjects who transition to and from higher-risk BMI categories during follow-up. Indeed, there is evidence to suggest that, among individuals having recently experienced a life-threatening event related to cardiovascular disease (e.g., myocardial infarction or stroke), those with excess weight display healthier trajectories of lifestyle and weight changes than normal weight individuals [39]. Although a recent study determined that repeated measures of BMI did not change mortality estimates [40], future analyses should incorporate more complex analytical methods and life-course conceptual models to address this potential bias [39].

We decided to not control for weight loss due to pre-existing illness (or what is commonly referred to in the obesity literature as “reverse causation” or “washout”). Certain studies have found that by excluding deaths that occur in the first few years of follow-up, mortality risks associated with the excess weight BMI categories become much higher. It is hypothesized that this is due to the greater presence of pre-existing illnesses amongst those in the normal weight BMI category at baseline, which in turn lowers their life expectancy compared to those with an excess body mass [41]. A recent mortality study using the same cohort as our study found that results by BMI category were not significantly affected when deaths occurring within the first four years of follow-up were excluded from analysis [5]. Furthermore, studies that actually assess body weight prior to baseline demonstrate no clear association between pre-existing weight loss and subsequent development of cancer or other chronic diseases commonly perceived to be associated with both weight loss and increased mortality risk. Methods to address confounding by illness-related weight loss, such as excluding deaths, may additionally introduce new biases since they will most likely also be excluding large numbers of subjects whose weight loss is not related to illness [42].

We also combined cycles of the CCHS in order to obtain greater stability in estimates of BMI prevalence and HRQL by BMI category. However, this approach may obscure possible trends occurring over the time period covered by the combined cycles.

Self-reports of height and weight, which are used to calculate HR and to estimate age-sex-BMI category-specific HRQL estimates, systematically underestimate true weight and overestimate true height. The results of this study may not reflect health expectancy estimates according to BMI calculated using measured height and weight. In order to assess the extent of this bias, we conducted sensitivity analyses using a previously published algorithm [25] that adjusts self-reported BMI values so that they more closely approximate directly-measured values. While estimates calculated using this correction factor did not appreciably differ from uncorrected estimates, the HR for obese class 1 went from being marginally significant at the p < 0.05 threshold to being no longer significant at that threshold. In addition, the LE calculated for obese class 1 was higher than that of normal weight men and women but was not significant at the p < 0.01 level. This leads us to conclude that the association between obese class 1 men and women for decreased mortality risk and increased LE is a weak one and should be interpreted with some caution.

The current study also did not report on health expectancy using other measures of body weight (e.g., waist circumference, skinfold thickness, waist-to-height ratio), as none of these alternatives are available in the National Population Health Survey. These measures can provide more accurate representations of adiposity, and consequently health risk, by distinguishing between lean and fat body mass [37,43].

Since the goal of this study was to describe HALE by BMI at the population level, we did not adjust for any socioeconomic or behavioral factors. We did however assess the effect of tobacco smoking on our study results by introducing a smoking covariate (“ever” versus “never” tobacco smoker) into the adjusted mortality hazard ratio model. The smoking covariate did not significantly change the estimates and was therefore not included in this report. This finding is consistent with previous recent research on mortality by BMI category using NPHS study data [5], although reduced sample size in that study may have decreased the power to detect effects. Since our study is based on the same cohort, we may have experienced the same decrease in power. Majer *et al.*, using a much larger sample size (n = 66,331), found that daily smokers had a lower LE compared to never-smokers of the same BMI category. However, their study also observed that patterns of life expectancy between each BMI category did not change appreciably when stratifying by smoking status [16]. Whether factors such as tobacco smoking, alcohol consumption, physical activity level, education, and income moderate observed levels of HALE by BMI group could be examined in future studies. It would also be important to consider that some of these factors commonly treated as confounders are part of the causal web surrounding behavior, BMI, and health, and a strategic approach considering potential mechanisms underpinning the observed relationships should take this into account.

## Conclusions

Consistent with other reports in the literature, our study among Canadian adult males and females found that those who are overweight or moderately obese have a higher or equivalent life expectancy respectively than those who have a normal weight. We also found a progressive increase of proportion of LE spent in nonoptimal health as BMI progresses beyond normal weight in both sexes for the Canadian adult population. Based on our results, which demonstrate an increased proportion of life expectancy spent in nonoptimal health for all overweight and obese individuals, as well as a loss of life expectancy for obese class 2+ individuals, our study reinforces the public health message which states that public health interventions should continue to focus on preventing normal weight and overweight persons from becoming obese. It should be noted that this study provides descriptive, summary estimates of the mortality and morbidity experience of Canadians by BMI category and, as such, does not add to our knowledge regarding the causal relationship between excess and insufficient body weight on the one hand and life expectancy and healthy life expectancy on the other. As such, further research is needed to identify which modifying behaviors and biological factors may be driving these results, including some of the differences between the sexes observed in our study. Of particular interest would be an investigation of the role these factors play in the heterogeneity of LE and HALE results in the obesity subcategories (e.g., life expectancy results in both sexes and health expectancy results in men). Finally, more enquiry is also needed to better understand the strengths and limitations of assessing life expectancy and health expectancy by body weight with currently available measures, and in particular with the BMI measure.

## Competing interests

The authors declare that they have no competing interests.

## Authors’ contributions

All authors contributed to the design of the study. CS oversaw the research. CS led the drafting and revision of the manuscript with contributions from LL and HO and input from all authors. LL oversaw statistical analysis and preparation of results with input from all authors. All authors read and approved the final manuscript.

## Supplementary Material

Additional file 1 Formulae for attributing mortality risk to the Canadian population by BMI category. Click here for file
