# Supplementary material for: Comparing life expectancy and health-adjusted life expectancy by body mass index category in adult Canadians: a descriptive study
Source: Popul Health Metr. 2013 Nov 19;11:21. doi: 10.1186/1478-7954-11-21 (PMC3842774; doi:10.1186/1478-7954-11-21)
Supplement: Additional file 1 — Formulae for attributing mortality risk to the Canadian population by BMI category. [file 1478-7954-11-21-S1.pdf]

Appendix: Formulae for attributing mortality risk to the Canadian population by BMI category

By taking into account BMI prevalence (Q_i) and the relative risk of dying (RR_{ij}) for each BMI category relative to the normal weight category, the age-specific mortality rates for the total Canadian population (R_t) are decomposed into mortality rates by BMI class. Based on the assumption that the sum of BMI prevalence across all BMI categories (N) is equal to 1 ($\sum_{i=1}^{N} Q_i = 1$) for each sex-age-group combination, the mortality rates for the reference category (R_j, normal weight) and for all other BMI categories (R_i) are expressed as

$$R_j = R_t \left\{ (1 - \sum_{i \neq j}^{N} Q_i) + \sum_{i \neq j}^{N} RR_{ij} Q_i \right\}^{-1} \qquad (5)$$

$$R_i = RR_{ij} R_t \left\{ (1 - \sum_{i \neq j}^{N} Q_i) + \sum_{i \neq j}^{N} RR_{ij} Q_i \right\}^{-1} \qquad (6)$$
